# The Effect of Anxiety on Regional Brain Volumes in the National Alzheimer’s Coordinating Center Uniform Data Set

**DOI:** 10.1093/geroni/igaa057.1197

**Published:** 2020-12-16

**Authors:** Shanna Burke, Tan Li, Adrienne Grudzien, Christopher Barnes, Kevin Hanson, Steven DeKosky

**Affiliations:** 1 Florida International University, Miami, Florida, United States; 2 University of Florida, Gainesville, Florida, United States; 3 University of Florida College of Medicine, Gainesville, Florida, United States

## Abstract

Anxiety has been associated with greater risk of Alzheimer’s disease (AD) and existing research has identified structural differences in regional brain tissue in anxious compared to healthy samples, but results have been variable and somewhat inconsistent. We sought to determine the effect of anxiety on regional brain volumes by cognitive and apolipoprotein e (APOE) e4 status using data from a large, national dataset. A secondary analysis of the National Alzheimer’s Coordinating Center Uniform (NACC) Data Set was conducted using complete MRI data from 1,371 participants (mean age: 70.5; SD: 11.7). Multiple linear regression was used to estimate the adjusted effect of anxiety (via the Neuropsychiatric Inventory Questionnaire) on regional brain volumes through measurement of 30 structural MRI biomarkers. Anxiety was associated with lower total brain and total cortical gray matter volumes and increased lateral ventricular volume (p<.05). Lower mean volumes were also observed in all hippocampal, frontal lobe, parietal lobe, temporal lobe, and right occipital lobe volumes among participants who reported anxiety. Conversely, greater ventricular volumes were also correlated with anxiety. Findings suggest that anxiety is associated with significant atrophy in multiple brain regions and ventricular enlargement, even after controlling for intracranial volume and demographic covariates. Anxiety-related changes to brain morphology may contribute to greater AD risk.

